# A novel nonsense mutation in the *NDP* gene in a Chinese family with Norrie disease

**Published:** 2010-12-08

**Authors:** Deyuan Liu, Zhengmao Hu, Yu Peng, Changhong Yu, Yalan Liu, Xiaoyun Mo, Xiaoping Li, Lina Lu, Xiaojuan Xu, Wei Su, Qian Pan, Kun Xia

**Affiliations:** 1 The State Key Laboratory of Medical Genetics of China, Central South University, ChangSha, China; 2 College of Medicine, Qingdao University, Qingdao, China; 3 Experimental Center, The People’s Hospital of Guangxi Zhuang Autonomous Region, NanNing, Guangxi, China; 4 School of Biological Science and Technology, Central South University, ChangSha, Hunan, China

## Abstract

**Purpose:**

Norrie disease (ND), a rare X-linked recessive disorder, is characterized by congenital blindness and, occasionally, mental retardation and hearing loss. ND is caused by the Norrie Disease Protein gene (*NDP)*, which codes for norrin, a cysteine-rich protein involved in ocular vascular development. Here, we report a novel mutation of *NDP* that was identified in a Chinese family in which three members displayed typical ND symptoms and other complex phenotypes, such as cerebellar atrophy, motor disorders, and mental disorders.

**Methods:**

We conducted an extensive clinical examination of the proband and performed a computed tomography (CT) scan of his brain. Additionally, we performed ophthalmic examinations, haplotype analyses, and *NDP* DNA sequencing for 26 individuals from the proband’s extended family.

**Results:**

The proband’s computed tomography scan, in which the fifth ventricle could be observed, indicated cerebellar atrophy. Genome scans and haplotype analyses traced the disease to chromosome Xp21.1-p11.22. Mutation screening of the *NDP* gene identified a novel nonsense mutation, c.343C>T, in this region.

**Conclusions:**

Although recent research has shown that multiple different mutations can be responsible for the ND phenotype, additional research is needed to understand the mechanism responsible for the diverse phenotypes caused by mutations in the *NDP* gene.

## Introduction

Norrie disease (ND; OMIM 310600), a rare X-linked recessive disorder, is characterized by congenital blindness and, occasionally, mental retardation and hearing loss. Degenerative and proliferative changes are observed in the neuroretina of ND patients and lead to early-onset blindness. More complex phenotypes are included in some ND cases, such as microphthalmia, growth failure, and seizures [1,2]. ND is caused by mutations in the *NDP* gene, which is located on chromosome Xp11.1. The *NDP* gene contains three exons, but only the second and the third exons are translated [3]. Norrin—a 133-amino acid protein encoded by the *NDP* gene—plays a critical role in the norrin-FZD4 signaling system, which is involved in the vascular development of the eye and ear [4]. More than 75 disease-causing mutations have been identified in ND patients [5-9]. Lack of norrin expression, truncated norrin, or mutations affecting the Cys residues involved in the conserved motif are much more likely to cause ND. However, the mechanism responsible for the variety of effects caused by norrin mutants is not entirely clear.

Here, we report a family possessing a novel nonsense mutation in the *NDP* gene, accompanied by severe presentation of ND in the three affected male patients. This is the first report of a Chinese family with three affected males with the *NDP* mutation. The proband is a 20-year-old male afflicted with symptoms typical of ND, including blindness, epileptic seizures, and degenerative neuropathy characterized by mental retardation. A computed tomography (CT) scan of his brain indicated remarkable cerebellar atrophy and the fifth ventricle between the left and right transparent septum, which is not common in human brains.

## Methods

The 26 patients studied here comprise three generations of a Chinese family from Shandong Province. The proband, III:5, was born in 1989 after a normal pregnancy. When he was two months old, he was sent to a local hospital where he was diagnosed with congenital cataracts. His medical record described the following characteristics: cloudy cornea in both eyes, lens opacities, leukoplakia of the cornea in both eyes, and posterior synechiae in the left eye. The right pupil could not be seen, and the diameter of the left pupil was 2 mm. In 2008, the proband and his two affected relatives were examined in further detail, and a CT scan of the proband’s brain was performed. Considering the possibility of haploinsufficiency, a magnetic resonance imaging (MRI) scan of the carrier III:6’s brain was performed as well.

Genomic DNA was extracted from the peripheral blood using the standard phenol/chloroform method and was stored at −20 °C. The study complied fully with the Tenets of the Declaration of Helsinki and was confirmed by the Ethics Board of the State Key Laboratory of Medical Genetics of China. Informed consent was given by all members of the family before testing. Because the proband was unable to communicate with us, his informed consent was obtained from his parents.

Microsatellite DNA markers were mapped to genes on the X chromosome by polymerase chain reaction (PCR) with 17 fluorescent microsatellite markers (ABI prism linkage mapping set version 2.5: DXS1060, DXS8051, DXS987, DXS1214, DXS1068, DXS993, DXS991, DXS986, DXS990, DXS1106, DXS8055, DXS1001, DXS1047, DXS1227, DXS8043, DXS8091, and DXS1073). To perform the PCR, a calculated master mix was created and then divided to several tubes equally. Each tube’s ingredients were 50 ng genomic template DNA, 0.5 μl PCR 10× buffer, 0.1 μl dNTP mix (2.5 mM), 0.06 μl each of forward and reverse primers, 0.6 μl MgCl_2_ (15 mM), and 0.05 U of AmpliTaq Gold (Applied Biosystems, Foster City, CA); the solution was brought to 5 μl with deionized water. Thermal cycling was conducted as follows: 95 °C for 12 min; 15 cycles of 94 °C for 30 s, 63 °C for 1 min with a decrease of 0.5 °C per cycle, and 72 °C for 1 min 50 s; 24 cycles of 94 °C for 30 s, 56 °C for 1 min, and 72 °C for 1 min 50 s; and a final extension step of 72 °C for 15 min (GeneAmp 2720, Applied Biosystems).

PCR products were analyzed on an ABI 3100 automated sequencer (Applied Biosystems). GS400 was used as an internal size standard and was run in the same lane as the markers. GENESCAN and GENOTYPER software were used to determine the size of the alleles. Subsequently, an additional 12 fluorescent markers from the Human Genome Database and Marshfield Database were analyzed for fine-scale mapping (DXS8090, DXS8015, DXS8012, DXS8085, DXS8035, DXS8080, DXS8054, DXS8083, DXS1003, DXS1039, DXS988, and DXS1204).

Two-point LOD scores were computed with the MLINK linkage analysis program (by Jurg Ott, version 5.2) [10]. All linkage analyses were performed using an X-linked recessive inheritance model with full penetrance in homozygotes and hemizygous males and a disease-allele frequency of 0.0001. Map distances were taken from the Marshfield Database, and haplotypes were manually reconstructed.

Mutation screening of the *NDP* gene, for all family members and for 100 healthy volunteers as normal controls, was performed in conjunction with X chromosome scans. PCR was used to amplify the region spanning exon 2 (forward primer: 5′-ATC CTG CCC TTT CCT TGA; reverse primer: 5′-AGC CTC ATT CTC CCA CAA), and exon 3 (forward primer: 5′-TGA GCC ACT GGT CTA ATC TA; reverse primer: 5′-CTC TCT CTG TCA ACA AGC AT) of the *NDP* gene. To perform the PCR, a calculated master mix was created and then divided to several tubes equally. Each tube’s ingredients were 1 μl (50 ng) genomic DNA, 1 μl (30 ng) of each primer, 1 μl 10× PCR buffer with MgCl_2_ (Roche Diagnostics Corporation, Indianapolis, IN), 0.05 μl (5 U) of AmpliTaq Gold (Applied Biosystems); the solution was brought to 10 μl with deionized water. The PCR products were purified with shrimp alkaline phosphatase (Fermentas International Inc., Burlington, Ontario, Canada) and exonuclease I (Fermentas) for 90 min at 37 °C to remove the phosphoryl groups. The samples were sequenced on an automated sequencer in both directions according to the manufacturer’s recommendations (ABI PRISM 3100 Genetic Analyzer, Applied Biosystems).

## Results

### Clinical findings

Three males from the Chinese family—the proband, his uncle, and his cousin—are affected by the disease, indicating that it might be inherited in an X-linked recessive manner.

**Figure 1:**
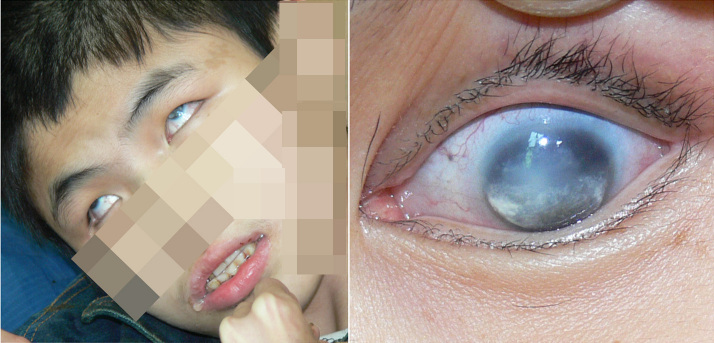
The proband, III:5, and his left eye. Cloudy cornea in both eyes, lens opacities, leukoplakia of the cornea in both eyes, and posterior synechiae in the left eye were observed. Further, the patient was suffered by seizures and mental disorders. The features of rolled up eyes and dribbling can be observed in this image.

Results of visual acuity examination of the proband were: no light perception in either eye, bilateral adherent leucoma of cornea, congenital cataract (Figure 1). Because the proband had severe mental retardation and epilepsy, the anterior chamber and posterior segment of his eye could not be examined. A B-ultrasonic scan showed that the optic axes are 21 mm in the right eye and 20 mm in the left eye and vitreous opacity. No retinal detachment was found.

**Figure 2:**
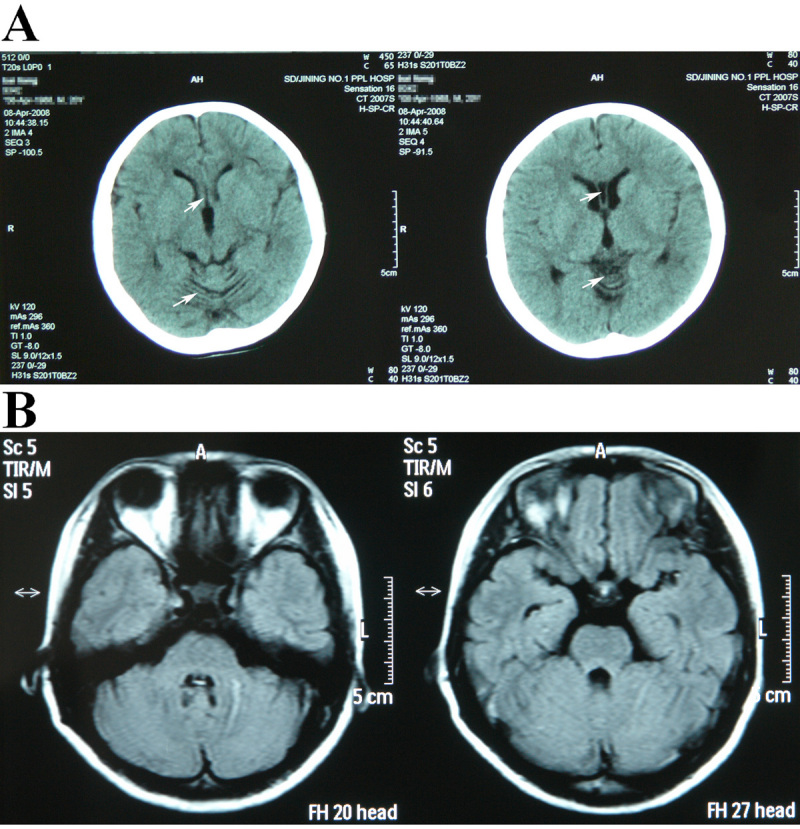
The CT scan and MRI scan results. **A**: CT scan of the proband’s brain; these images show cerebellar atrophy with widened and deepened sulci and permit observation of the fifth ventricle. Arrows show cerebellar atrophy is characterized by narrowed lobes and widened folds of cerebellar hemisphere and vermis; and the evolution of the cavity of septum pellucidum. **B**: MRI scan of carrier III:6’s brain; no abnormality was observed.

A CT scan of the proband’s brain (Figure 2) indicated that he had experienced cerebellar atrophy with widened and deepened sulci; the fifth ventricle was also observed on the scan. Cerebral density remained normal; no widening and deepening of the cerebral sulcus fracture were found; and the position of central structure had not changed.

The other two patients were also afflicted by epilepsy and progressive mental disorders, and can barely communicate with other people. Even with assistance, they are barely able to walk; however, they are more mobile than the proband, who can only be moved in a wheelchair. The conditions of all three patients have worsened with age.

No ND phenotype was found in the carriers. The MRI scan of carrier III:6’s brain showed no abnormality (Figure 2).

### Linkage analysis

The maximum LOD score for all 17 analyzed markers was 1.51 for DXS993 on chromosome Xq11.4. On the basis of this result, we analyzed 12 additional fluorescent markers in the flanking regions of DXS993 for fine-scale mapping. Of these, 10 yielded additional information; three of these markers (DXS8012, DXS8035, and DXS1003) had LOD scores greater than 2 (Table 1), which suggested that they were involved in the ND phenotype.

**Table 1:** Two-point LOD scores for microsatellite DNA markers.

		**Recombination fraction**					
**Marker**	**cM**	**0.00**	**0.1**	**0.2**	**0.3**	**0.4**	**0.5**
DXS8090	36.79	-∞	0.09	0.33	0.29	0.14	0.00
DXS8015	37.87	0.90	0.77	0.61	0.44	0.24	0.00
DXS8012	42.21	2.41	2.00	1.54	1.02	0.45	0.00
DXS993	42.21	1.51	1.23	0.92	0.58	0.21	0.00
DXS8085	42.75	0.90	0.77	0.61	0.44	0.24	0.00
DXS8035	43.83	2.41	2.00	1.54	1.02	0.45	0.00
DXS8054	45.87	1.51	1.23	0.92	0.58	0.21	0.00
DXS8083	46.54	1.51	1.23	0.92	0.58	0.21	0.00
DXS1003	47.08	2.41	2.00	1.54	1.02	0.45	0.00
DXS988	52.50	-∞	0.09	0.36	0.35	0.22	0.00
DXS1204	52.50	-∞	0.09	0.36	0.35	0.22	0.00

### Haplotype analysis and recombination mapping

**Figure 3:**
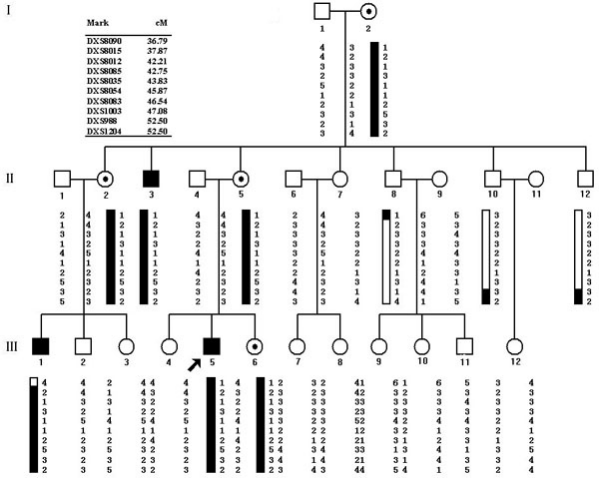
Pedigree of the study family and the haplotypes obtained from examining 12 microsatellite DNA markers on chromosome X. Solid symbols represent affected individuals and open symbols represent unaffected individuals. The arrowhead denotes the proband. Markers are listed in order from the centromere to the telomere. The affected haplotype is shown in rectangles*.*

All 12 fluorescent microsatellite markers flanking DXS993 were used to construct the haplotypes. Inspection of the haplotype transmission data (Figure 3) showed a telomeric recombination event between markers DXS8090 and DXS8015 in individuals II:8 and III:1, placing the disease-causing gene centromeric to marker DXS8090. Similarly, recombination events between loci DXS1039 and DXS988 occurred in individuals II:10 and II:12, indicating that the disease gene is telomeric to locus DXS988. Thus, in this family, the disease gene lies within a 15.71-cM region on chromosome Xp21.1-p11.22, bounded proximally by locus DXS8090 and distally by locus DXS988.

### Mutation screening

**Figure 4:**
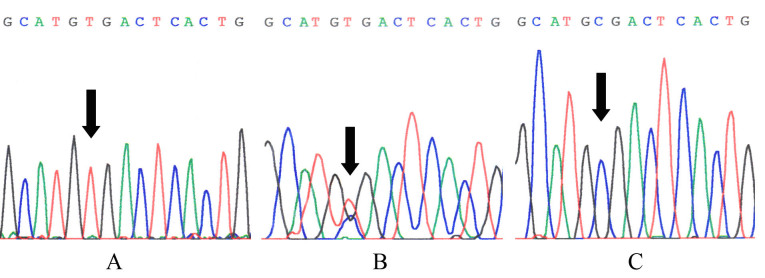
Partial sequences of *NDP* exon 3 from the ND patient III:5 (**A**; homozygous for the c.343C>T genotype), carrier III:6 (**B**; heterozygous for the genotype), and control (**C**). The black arrows show the mutation CGA > TGA in the proband and a carrier, altered the arginine codon to a termination codon, but this mutation was not found in normal controls.

The three affected patients (II:3, III:1, and III:5) were homozygous for a novel nonsense mutation, c.343C>T; four other individuals (I:2, II:2, II:5, and III:6) were heterozygous for this genotype (Figure 4). This transition causes an Arg115Term exchange and results in the production of a nonfunctional truncated norrin protein. The distribution of carriers and non-carriers in this pedigree conforms to the predictions of the haplotype analysis and recombination mapping. The c.343C>T mutation was not found in the controls.

## Discussion

ND is rarely reported in China. Here, we have used CT scans, genome scans, and mutation screening to confirm the characteristics of ND and diagnose ND according to Warburg’s criteria [11]. Mental disturbances have also been described in other ND patients [12,13]. However, previous reports did not describe motor disorders or cerebellar atrophies as severe as those documented here. These results may provide more data for research and molecular diagnosis of the disease.

The *NDP* gene maps to Xp11.1, spans 28 kb, and contains 3 exons. Exon 1of the *NDP* gene contains only the 5′ untranslated region; the first 58 residues of the open reading frame are in exon 2, while codons 59–133 of the open reading frame and the 3′ untranslated region are in exon 3 [3]. ND is ascribed to mutations of the *NDP* gene, which can also cause X-linked familial exudative vitreoretinopathy (XL-FEVR) [14-16]. XL-FEVR is a heterogeneous vitreoretinal disorder characterized by peripheral vitreous opacities, subretinal and intraretinal exudates, and retinal traction due to failure of peripheral retinal vascularization [17].

*NDP* encodes norrin, a member of the cystine knot growth factor family [18]. It is a secreted cysteine-rich protein that has 133 amino acids. The cystine knot domain of norrin spans from codon 32 to codon 133, which is thought to play an essential role in neurologic interactions [19]. The cysteine residues at codons 39, 65, 69, 96, 126, and 128 have been found to be responsible for the cysteine-knot formation. Three disulfide bridges between codon 39 and 96, 65 and 126, and 69 and 128 are involved in the tertiary structure of norrin [5]. Most mutations of the *NDP* gene associated with ND or XL-FEVR are related to this domain, including R115L (c.344G>T) and R109Term (c.325C>T), which cause XL-FEVR and ND, respectively [20]. Thus far, all patients with nonsense mutations were diagnosed with ND disease.

The novel mutation in exon 3 at codon 115 altered the arginine codon to a termination codon. Hence, the last 18 amino acids of the wild-type norrie protein would lost by the resulting protein, which contains cysteine residues and disulfide bridges involved in the cysteine knot. The R115Term mutation might considerably alter the protein structure and severely disrupt neurologic interactions related to the cystine knot domain.

The function of norrin is not entirely clear; however, research has shown that it may be involved in the development of the neuroectoderm or in regulation of neural cell proliferation [21]. Norrin has been identified as a specific ligand for frizzled-4 (FZD4; OMIM 604579), a presumptive Wnt receptor. Interacting with the cysteine-rich domain (CRD) of FZD4, norrin can activate the Wnt/β-catenin pathway by inducing FZD4- and LRP (OMIM 107770)-dependent activation, affecting vascular development in the ear and eye [4]. Moreover, norrin plays an important role in retinal vasculogenesis and in neural cell differentiation and proliferation [22,23]. Additionally, evidence has shown that the neuroprotective properties of norrin are independent from its effects in vascular development [24]. This may explain why degenerative neuropathies such as seizures and mental disorders were observed in the affected individuals examined in this study.

However, the mechanism by which mutations in the *NDP* gene cause the complex phenotypes of ND is still unclear. Further, the case presented here supports Schuback’s assumption that new phenotypic expressions of the disease may be identified [6]. This may result from complex epigenetic factors that interact and influence the physiologic and neurodevelopmental expression of the ND phenotype [6]. More data are required to further the understanding of the diverse and variable effects of norrin mutations.
